# Forced oscillation response of the dynamic surface tension of molten titanium

**DOI:** 10.1371/journal.pone.0338206

**Published:** 2025-12-04

**Authors:** Zhiyong Yu, Wenjun Li, Xiaowei Dai, Yang Yang, Yangyang Zhao, Boxue Song

**Affiliations:** 1 School of Intelligent Manufacturing, Putian University, Putian, Fujian, China; 2 School of Artificial Intelligence, Putian University, Putian, Fujian, China; 3 College of Computer and Data Science, Putian University, Putian, Fujian, China; 4 School of Physics and Electronic Science, East China Normal University, Shanghai, China; UNIPI: Universita degli Studi di Pisa, ITALY

## Abstract

**Background:**

This study employed the molecular dynamics simulation method to systematically investigate the dynamic response behavior of the molten titanium liquid-vapor interface under high-frequency (50 GHz) and large-amplitude (5%) transverse mechanical cyclic impact.

**Methods:**

Based on the theory of driven-damped oscillators, we analyzed the steady-state forced oscillation characteristics of dynamic surface tension. Through frequency analysis, the dependence of the system response on the impact frequency was revealed. And by using the liquid stratification method, we investigated the space-time correlation characteristics of the bulk and surface atomic dynamics.

**Results:**

This study mainly found that the average value of dynamic surface tension increased by 7% compared to the equilibrium state, confirming that high-frequency mechanical impact has a regulatory effect on surface tension. Meanwhile, the peak and valley of instantaneous fluctuations reached 14% and 5% of the equilibrium state respectively, presenting a significant nonlinear oscillation characteristic. Theoretical analysis indicates that there is a coupling effect between the generalized natural frequency and the damping constant. Experimental observations show that the atomic dynamics behavior of the outermost layer is significantly different from that of the bulk liquid.

**Conclusion:**

This study has deepened our understanding of the dynamics of the liquid-vapor interface under extreme conditions. It provides new theoretical basis for understanding the multi-scale behavior of liquid metals and has guiding significance for the surface control of high-frequency mechanical impacts and industrial applications.

## 1 Introduction

In advanced manufacturing, metallurgy, and new energy fields, the instantaneous dynamic changes of the surface tension of molten metals are of great significance [1–3]. The dynamic spatiotemporal evolution of surface tension has a significant impact on the capillary fluctuations during the physical and chemical property changes of materials [4–6], and also plays an important role in the synthesis and preparation of materials [7–8]. We used classical molecular dynamics to simulate the ultrafast evolution behavior of titanium (Ti) melt at high resolution and successfully captured the ultrafast evolution process of dynamic surface tension in molten liquids that is difficult to directly observe in experiments. This process has a direct impact on the microscopic mechanism of dynamic forced oscillation of surface tension. Although experiments have successfully detected ultrafast atomic density fluctuations induced by femtosecond laser pulses through ultrafast electron diffraction technology [9], the understanding of the ultrafast dynamic characteristics of liquid surfaces under shock loading [10–12] is still insufficient. Therefore, further in-depth exploration is still needed.

For the molecular dynamics (MD) simulation of liquid systems, its essence is studying the response behavior of the system to changes in external temperature and pressure. Under constant temperature conditions, the common pressure calculation method is the pressure expression based on the virial definition [13]. Further, the surface tension integral path calculation methods developed based on virial theorems [14,15] or fluctuation-dissipation theorems [16,17], such as the Irving-Kirkwood (IK) [18,19] and Harashima (H) [20,21] methods. They have become important tools for studying interface properties from the microscopic scale. The quantum molecular dynamics (QMD) method [22,23], especially the Ab Initio Molecular Dynamics (AIMD) based on density functional theory [24,25], has shown unique advantages in studying the surface tension of complex systems such as metals and silicates [26–28]. Being able to accurately describe electronic effects and chemical bond behaviors is the core [29]. Due to the inclusion of quantum effects of the electronic structure in the kinetic evolution process [30], QMD provides a reliable means to reveal interface reactions involving the formation and breaking of chemical bonds (such as the dissociation of water molecules [31]). This is particularly important in extreme conditions such as high temperature [32], high pressure [33], and reactive systems [34]. MD and QMD simulations not only provide a unique microscopic perspective but also form an effective complement and contrast with continuum theory models [35]. This paper continues this research paradigm in terms of methodology, aiming to leverage the advantages of MD simulation to systematically explore the dynamic response behavior of surface tension under the aforementioned extreme non-equilibrium conditions.

The motivation for this study stems from the recent breakthroughs in ultrafast manipulation of interfaces in condensed matter, particularly the achievements of precisely controlling interface structure and dynamics using femtosecond pulses [36–39]. Yang et al. [40,41] demonstrated through atomic-scale simulations that under the action of a single femtosecond laser pulse, the surface tension of a metal can undergo ultrafast modulation within picosecond timescales, with its fluctuation amplitude even exceeding 20% of the equilibrium value. Further analysis revealed that the laser-induced shock waves would trigger significant surface stress fluctuation effects. This stress redistribution in the non-equilibrium state provides a new perspective for understanding the dynamics of interfaces. When studying capillary phenomena and curvature-related surface tension [42,43], the liquid-vapor interface is usually analogized to an elastic membrane system [44,45]. This theoretical framework has gained wide acceptance. However, when the system is subjected to extreme loading conditions at picosecond or even shorter time scales, the response behavior of the liquid surface under dynamic driving has significantly deviated from the description of classical theories. Currently, the mechanical response mechanism of dynamic liquid surface tension under extreme non-equilibrium conditions is still poorly understood, and experimental verification is also scarce. In particular, there is no consensus on this key issue. At such extreme spatial and temporal scales, does the behavior of the liquid interface still follow the basic laws of classical elasticity mechanics? What new physical images will its non-equilibrium response exhibit? In-depth exploration of these questions will drive the breakthrough development of interface physics theories under extreme conditions.

To deeply explore the issues mentioned in the previous text, this paper uses molecular dynamics simulation methods to conduct an in-depth study on the dynamic characteristics of the titanium (Ti) liquid surface under the action of transverse mechanical cyclic impact loads. In the previous research work [46], we have found that the dynamic surface tension response behavior of the lead (Pb) liquid is in good agreement with the classical theory. This study found that the dynamic surface tension of the titanium (Ti) liquid still conforms to the classical theory of driving damping oscillators on the whole. The surface tension of the titanium (Ti) liquid exhibits a clear forced damping oscillation response behavior, but it is different from the surface tension of the lead (Pb) liquid at high frequencies. The 50 GHz loading condition adopted in this study matches the characteristic time scale of non-equilibrium processes such as femtosecond laser shock [47] and ultrafast phonon excitation, providing an effective research approach for revealing the transient interfacial dynamics mechanism at the atomic scale. Based on the experimental observation results of the simulation experiments, we speculate that these differences may be related to the collective dynamic characteristics of the atomic layer on the surface of the titanium liquid. At high frequencies, this difference may be manifested in the relaxation time scale, local structural order degree, and atomic packing density of the two liquids. In order to deeply understand this phenomenon, we introduced two key generalized elastic characteristic parameters, namely generalized natural frequency and generalized damping coefficient, into the theoretical model. Through the quantitative analysis of these two characteristic parameters, not only can the dynamic response characteristics of the liquid surface be described, but it is also possible to provide a new research perspective for exploring the intrinsic time scale of the atomic spatiotemporal correlation function of the liquid.

## 2 Simulation method

We employed the molecular dynamics simulation method to investigate the dynamic behavior of the liquid-titanium-vapor interface (LVI) at the melting point temperature (*T*_*m*_). This study employed a large-scale atomic/molecular parallel simulator (LAMMPS) to conduct molecular dynamics simulations. During the melting-equilibrium stage and the mechanical cyclic impact load stage, the simulation system contained 33,264 titanium atoms, with the interatomic interactions described by an embedded atom potential. The simulation box size was 83 × 92 × 395 Å³, with a block of titanium approximately 80 Å thick placed at the center along the *z*-axis. It formed two parallel liquid-vapor interfaces. The lattice constant was set to 2.951 Å, and the structure was *hcp*. Periodic boundary conditions were applied in the *x*, *y*, and *z* directions. The cutoff radius for atomic interactions was 2.0 Å, and the neighbor list was updated every 1 step, with atom ordering disabled. We calculated the potential energy (pe/atom) and virial stress (stress/atom) of each atom, with bin sizes of 0.01 Å for spatial partitioning. The simulation time step was 0.001 ps. Thermodynamic information was output every 1 ps, including temperature, pressure, total energy, volume, *p*_*xx*_, *p*_*yy*_, *p*_*zz*_, and the boundary sizes of each direction of the box. Variables such as total energy, volume, box length, and stress components were defined for analysis. The restart file was saved every 100 ps, the particle velocities were initialized according to the Maxwell-Boltzmann distribution, and the system’s net momentum was removed. In each integration step, the linear momentum in the three directions was retained. The trajectory output and *NVT* ensemble settings were completed according to the standard procedure. We heated the Ti system to 2000 K at a temperature step of 1 K/ps (by setting the simulation temperature to 1 K/ps) to prepare the liquid Ti, and then cooled it to 1531 K at a temperature step of 10 K/ps (by setting the simulation temperature to 10 K/ps) using a Nose-Hoover thermostat to set the equilibrium temperature *T* = *T*_*m*_ = 1531 K. The equilibrium temperature was consistent with the experimental value of 1500 K at this melting point [48]. The *NVT* simulation time of the system exceeded 50 nanoseconds, ensuring that the LVIs system reached the thermodynamic equilibrium state.

During the mechanical cyclic impact load stage, the above-end coordinate was extracted and the simulation was continued. Apart from the above parameters, a layered temperature control method [49] was introduced. This technique divides the system along the *z*-axis into multiple independent temperature-controlled layers with a thickness of 8 Å (parallel to the *xy* plane). The temperature of each layer is precisely controlled at 1531 K, effectively avoiding the local thermal disturbances that might be caused by traditional uniform temperature control [50]. This method has previously been successfully applied to the research on high thermal conductivity thermal management of metal materials. Following the method in reference [46], we fixed the center of the liquid block region, thereby accurately capturing the collective flow velocity of the system during the periodic loading process. Additionally, variables *f*(*t*) related to the loading of the titanium system and initial conditions were set. The specific implementation of the input file and program can be found at the following website address (https://github.com/windrunners/Ti-DST). In these files, “copy.1.file” contains the complete details of the melting–equilibrium stage, and “copy.2.file” contains the complete details of the mechanical cyclic impact load stage. The detailed loading process is described as follows.

**Fig 1:**
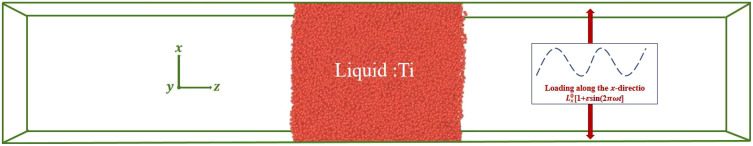
This figure illustrates the schematic diagram of the non-equilibrium molecular dynamics (MD) simulation of the titanium (Ti) liquid at its melting point. The simulation is carried out by applying mechanical cyclic loads along the *x*-axis. The simulation box includes the liquid phase and the vapor phase, as well as two liquid surfaces parallel to the *xy* plane.


Based on the equilibrium state of the molten titanium (Ti) system, we applied periodic cyclic loads *f*(*t*) along the normal direction of the liquid-vapor interface (LVI) using the molecular dynamics (MD) method to simulate and study the dynamic response of the system. After sufficient relaxation, the system reached a steady-state oscillation state. On this basis, we accurately measured the dynamic surface tension and systematically calculated the microscopic thermodynamic parameters of the LVI. In the non-equilibrium molecular dynamics simulation, the simulation system was subjected to periodic deformation regulation along the *x*-axis in the form of $L_{x}(t)=L_{x}(\text{0})\times f(t)$ (as shown in Fig 1). The initial simulation box size along the *x*-axis was $L_{x}(\text{0})$ = 81 Å. The cyclic load used in this study was applied in the form of a sine oscillation function, and its mathematical expression is as shown in equation (1).

f(t)=1+εsin(2πωt)=1+εsin(2πt/C) (1)

This study applies cyclic loading amplitudes *ε* and frequencies *ω* (*ω* = 1/*C*, where *C* is the loading cycle time) to the system to impose periodic deformations. The atomic coordinates and simulation box size are adjusted every 4000 molecular dynamics (MD) steps. When the system undergoes stretching or compression along a certain axis, the atomic coordinates in that direction will be scaled synchronously to match the change in the box size. The effects of different cyclic loading conditions were investigated, including four loading frequencies (50, 25, 5, and 1.25 GHz, corresponding to periods of 20, 40, 200, and 800 ps) and three loading amplitudes (1%, 3%, and 5%). The specific parameters are detailed in Table 1. Through this multi-parameter simulation scheme, the influence laws of cyclic loading characteristics on the mechanical behavior of the material were systematically studied.

**Table 1:** This table presents the simulation parameters of the non-equilibrium state molecular dynamics (MD) model for the molten titanium (Ti) liquid-vapor system under periodic loading. Here, the periodic loading is described by Equation (1). The main parameters of this equation include the loading amplitude *ε*, the loading frequency *ω*, and the duration of a single loading cycle *C*. Additionally, the table also lists the total duration *t*_NEMD_ of the non-equilibrium state molecular dynamics simulation, the duration *t*_steady_ after the system reaches steady-state oscillation, the number of steady-state cycles *n*_cyc_ used for calculating the dynamic surface tension, and the duration *t*_trans_ of the transition stage before the system reaches steady state.

ε(%)	*C*(ps)	ω(𝐆𝐇𝐳)	$t_{𝐍𝐄𝐌𝐃}^{𝐓𝐢}(𝐧𝐬)$	$t_{𝐬𝐭𝐞𝐚𝐝𝐲}^{𝐓𝐢}(𝐧𝐬)$	$n_{𝐜𝐲𝐜}^{𝐓𝐢}(𝐧𝐬)$	$t_{𝐭𝐫𝐚𝐧𝐬}^{𝐓𝐢}(𝐧𝐬)$
1	20	50	180	110	300	70
3	20	50	380	290	900	90
5	20	50	330	260	300	70
1	40	25	160	130	550	50
3	40	25	300	230	300	70
5	40	25	300	300	300	60
1	200	5	180	90	150	90
3	200	5	270	200	150	70
5	200	5	380	320	60	60
1	800	1.25	460	360	40	100
3	800	1.25	150	90	30	60
5	800	1.25	360	290	60	70

In the simulation, we employed the embedding atomic method (EAM) potential function developed by Mishin et al. for aluminum-titanium (Al-Ti) alloys [51]. This potential function predicted a titanium melting point of 1531 K in the simulation of crystal-melt coexistence, which is in good agreement with the experimental value of 1500 K [52]. This potential function demonstrates excellent applicability in the study of titanium-based systems. Its application outcomes mainly include successfully revealing the complex microscopic structural changes under the atomic configuration of the solid-liquid interface [53,54], accurately characterizing the transport properties of liquid titanium [55], and systematically elucidating the regulatory mechanism of cooling rate on the glass formation and crystallization behavior of Ti-Al alloys [56,57]. Particularly notable is that the structural evolution characteristics of the Ti-Al alloy during cooling obtained by this potential function simulation are highly consistent with the experimental observations [58]. This important discovery strongly validates its reliability in describing the phase transformation behavior of titanium-based systems. The above research results not only enrich the theoretical basis of titanium-based materials but also provide reliable methodological support for the molecular simulation of the surface properties of liquid titanium in this study.

## 3 Calculation method

This section systematically presents the calculation methods for the key thermodynamic parameters of the titanium liquid-vapor system under periodic cyclic loading. To ensure the reliability of the simulation results, we specifically conducted a stability test for the system. In the *NVT* simulation using the Nose-Hoover heat bath, the system exhibited good temperature stability both in the equilibrium state and during the equilibrium stage of cyclic loading (100 ps). The temperature fluctuation range was always controlled within 1531 K ± 2 K, which met the standard requirements for molecular dynamics simulations of metal systems. In terms of pressure stability, by monitoring the pressure components in three directions (*p*_*xx*_, *p*_*yy*_, *p*_*zz*_), it was found that the mean values of *p*_*xx*_ and *p*_*yy*_ directions of pressure fluctuated around 0.009 GPa, while the mean value of *p*_*zz*_ direction remained stable within 0.04 GPa. The convergence characteristics of the pressure in each direction indicated that the system had reached a sufficient pressure equilibrium state, providing a reliable foundation for subsequent thermodynamic analysis. Based on non-equilibrium MD simulation, when the system reaches a steady oscillatory state, we established the following calculation framework for the physical quantities.

### 3.1 Dynamic interface spatiotemporal evolution distribution

We calculated the time-dependent dynamic interface distribution functions [59] during the loading process by counting the number of atoms within the discrete box *δ*z (*δ*z = 0.1 Å) during the relaxation time. These functions include the dynamic density distribution function, the dynamic pressure component distribution function, and the dynamic stress distribution function. Specifically, the dynamic coarse-grained density distribution function *ρ* (*z*, *τ*) defined in equation (2) was obtained through the following method. In the steady-state oscillation state, the average number of atoms in each discrete box during a single load cycle (*τ* ≡ *t* mod *C* and $\tau =\tilde{t^{\text{1}}}$) or ten load cycles (*τ* ≡ *t* mod 10*C* and $\tau =\tilde{t^{\text{10}}}$) during the relaxation time was coun*t*ed and divided by its volume (V=A×δz and *A* is the cross-sectional area of the box).


$$\rho (z,\tau )=\frac{{\left\langle N_{z}(\tau )\right\rangle }_{n_{cyc}}}{A\delta z}$$ (2)

Where, *N*(*z*,τ) represents the number of atoms located within the discrete box of the interval [*z, z+*δz] at time τ. ${\left\langle N_{z}(\tau )\right\rangle }_{n_{cyc}}\;$represents the average value of the particle number samples of $n_{\text{cyc}}$ cycles extracted from the steady-state oscillation, and the specific values are listed in Table 1.

In traditional equilibrium *NVT* simulations, the calculation of atomic stress typically employs the Kirkwood-Buff equation [46] method. Half of the pairwise interaction potential force contribution is summed with the atomic kinetic term to calculate the potential force stress tensor for each atom (in units of pressure × volume). The specific expression is $S_{i}^{\alpha \beta }$=-[${mv}_{i\alpha }v_{i\beta }+\frac{\text{1}}{\text{2}}\sum_{j=\text{1}}^{N_{n}}\left(r_{i\alpha }f_{i\beta }+r_{j\alpha }f_{j\beta }\right)$]. Here, *α* and *β* represent the *x*, *y*, and *z* directions, $v_{i}$ is the velocity of atom *i*, $r_{i}$ and $r_{j}$ are the positions of atom *i* and atom *j*, $f_{i}$ and $f_{j}$ are the interaction forces between atom *i* and atom *j*, and $N_{n}$ is the number of neighboring atoms of atom *i*. However, this method is only applicable to equilibrium systems and cannot accurately describe the melt-state Ti metal surface system under cyclic loading as we are studying. This is because in non-equilibrium conditions, the metal liquid system undergoes stretching motion, introducing collective velocity. This physical quantity does not exist in the thermal equilibrium state. The original method of Kirkwood et al. did not consider the influence of this collective velocity, which led to significant deviations in the calculation of atomic stress and pressure at the interface. To solve this problem, we adopt an improved method [40,41] to correct the stress tensor. The calculation is $S_{i}^{\alpha \beta }$*=-*$\left[{m[v}_{i\alpha }{(t){-u}_{\alpha }(r_{i},t)][v}_{i\beta }(t)-u_{\beta }(r_{i},t)]+(\sum_{j=\text{1}}^{N_{n}}\left[(r_{i\alpha }{(t)f}_{i\beta }(t)+r_{j\alpha }(t)f_{j\beta }(t\right)])/\text{2}\right]$. Here, $u_{\alpha }(r_{i},t)$ represents the flow velocity of the metal liquid at position $r_{i}$. By subtracting the contribution of the collective motion velocity from the kinetic term of the stress tensor, this method effectively eliminates the influence of load-induced collective motion on the calculation results, thus being applicable to calculating the pressure component and stress field distribution of non-equilibrium surface systems related to stretching.


$$p_{\alpha \beta }(z,\tau )=-\frac{{\left\langle \sum_{i}^{N_{i}^{N_{z}(\tau )}}S_{i}^{\alpha \beta }\right\rangle }_{n_{cyc}}}{A\delta z}$$ (3)

In equation (3), $p_{\alpha \beta }(z,\tau )$ represents the dynamic fine-grained pressure component along the normal direction of the interface (the *z*-axis), with the spatial fine-grained resolution *δz*. This physical quantity can be calculated by dividing the negative sum of all atomic stress tensors $S_{i}^{\alpha \beta }$ within the discrete box by the volume Aδz of the box. The calculation is $\;S(z,\tau )=\;p_{zz}(z,\tau )-[p_{xx}(z,\tau )+p_{yy}(z,\tau )]/\text{2}$. The dynamic fine-grained stress distribution S(z,τ) is defined as the difference between the normal stress component $p_{zz}(z,\tau )$ and the transverse stress component $[p_{xx}(z,\tau )+p_{yy}(z,\tau )]/\text{2}$ [60]. Based on the analysis of the dynamic state equation, it is shown that after deducting the contribution of the collective flow velocity, the dynamic component of $p_{\alpha \beta }(z,\tau )$ shows a significant positive correlation with the density ρ(z,τ). It is noteworthy that the three main pressure components (*xx*, *yy*, and *zz*) exhibit obvious isotropic characteristics. This indicates that the contribution of the configuration plays an important role in $\;p_{\alpha \beta }(z,\tau )$, directly influencing the stress distribution characteristics and the formation mechanism of the dynamic surface tension (or excess stress). It should be noted that there are various equivalent microscopic pressure components for calculating the stress distribution and surface tension (excess stress) in the current literature [60–62]. To ensure the uniformity of the calculation method, this study chooses to use the excess stress method for calculation.

### 3.2 Evolution of dynamic surface tension

For the liquid-gas interface system in a fluid static pressure equilibrium state [63], the surface tension $\gamma_{eq}$ (which is the surface excess stress in the equilibrium state) can be defined and calculated using the Kirkwood-Buff equation theory [46]. The specific expression is $\;\gamma_{eq}=\int_{z_{lo}}^{z_{hi}}S_{eq}(z)dz$. When the system is under dynamic loading conditions, the liquid surface tension will exhibit time dependence. At this time, it can be defined as the dynamic surface tension  γ(τ). Based on this theoretical framework, Likhtman and Lukyanov systematically studied the dynamic surface tension evolution behavior of non-equilibrium liquid droplets [64]. This provides an important theoretical basis for understanding the interface dynamics process.

We applied cyclic loading to the titanium (Ti) liquid system. This significantly altered the fluid static pressure conditions of the system, especially under higher load frequency *ω* and larger load amplitude *ε*. Based on our previous research [46], this study detailed the distribution of dynamic excess stress and the calculation method of surface tension *γ*(*τ*) (excess stress) under the conditions of a 50 GHz load frequency and a 5% load amplitude. We further extended this method to the titanium (Ti) liquid-vapor system. Specifically, at the *τ* moment, we clearly defined the stress distribution of the vapor phase $L_{v}(\tau )$ and the liquid phase $L_{l}(\tau )$ through the Gibbs interface (GDS) [46]. Based on the characteristic lengths along the *z* direction of the liquid phase and the vapor phase, the dynamic surface tension *γ*(*τ*) (or interface excess stress) was calculated according to equation (4).


$$\gamma (\tau )=\left[\int_{{\text{z}}_{\text{lo}}}^{{\text{z}}_{\text{hi}}}S(z,\tau )\text{dz}\right]-S_{l}(\tau )L_{l}(\tau )$$ (4)

Among them, the calculation range values $z_{hi}$ and $z_{lo}$ along the *z* direction of the system correspond to the upper and lower boundaries of the selected area, and their value range is approximately half of the total length of the z direction of the system [46]. It is particularly important to note that the determination and dynamic changes of $z_{hi}$ and $z_{lo}$ in this paper are closely related to the selection of the dynamic Gibbs separation surface (GDS) (for the detailed selection method of GDS, please refer to our previous work [46]). In the specific calculation process, the position of the GDS is determined by the density distribution ρ(z,τ), and the dynamic surface tension is calculated based on the stress distribution analysis method established in the literature [46]. For each dynamic liquid Ti’s GDS interface in a stable oscillation state, the position of the GDS at *τ* should be selected such that the excess particle number $N_{excess}(\tau )$ in each ρ(z,τ) is equal to zero. $N_{excess}(\tau )=N-\rho_{l}(\tau )A\;L_{l}(\tau )-\rho_{v}(\tau )AL_{v}(\tau )=\text{0}$. *N* is the total number of particles in the system, $\rho_{l}(\tau )$ is the number density of the bulk liquid phase, *A* is the cross-sectional area, $L_{l}(\tau )$ is the corresponding length of the bulk liquid phase along *z* defined by the GDS at *τ*, $\rho_{v}(\tau )$ is the number density of the bulk gas phase, and $L_{v}(\tau )$ is the corresponding length of the bulk gas phase along *z* defined by the GDS at *τ*. S(z,τ) is called the dynamic fine-grained stress distribution, and $S_{l}(\tau )$ represents the local stress of the liquid phase after experiencing the cyclic load of time *τ* in a homogeneous non-flowing hydrostatic state. This stress value is determined by calculating the average dynamic fine-grained stress distribution S(z,τ) within the bulk liquid, and the characteristic value of the platform area in the distribution curve of S(z,τ) is selected. For the vapor phase part $S_{v}(\tau )$, its stress distribution $S_{v}(\tau )$ is set to zero in equation (4) (since $S_{v}(\tau )$ of the vapor phase part is 0). Finally, the Simpson integration rule (Simpson rule) is used to numerically integrate equation (4), thereby obtaining the surface tension of the system.

## 4 Results and discussion

Under cyclic loading, the dynamic surface tension of the molten titanium (Ti) liquid-vapor system shows a rapid increase during the initial transient stage (lasting approximately several tens of nanoseconds). It is notable that the relaxation time *t*_trans_ of this transient stage exhibits significant frequency and amplitude dependence, as shown in Table 1. Once the system passes through the transient stage, the dynamic surface tension enters a stable periodic oscillation state. Through coarse-graining of the data from the steady-state stage (with a time resolution of approximately 2 ns), we found that the average dynamic surface tension converges to a constant value. This value is significantly higher than the reference surface tension value of 995.583(3) mN/m at the equilibrium state of the melting point (*T* = *T*_*m*_).

To reveal the dynamic change mechanism of the surface tension at the molten titanium liquid-vapor interface (LVI) under cyclic loading, we conducted research based on the forced damped oscillator model. The results show that although the average position of the driving oscillator remains in the initial equilibrium, the dynamic surface tension of the LVI system has significantly increased. This difference stems from the structural reconfiguration of the LVI as a multi-atomic system under the load, and the simple oscillator model cannot fully describe this dynamic change effect. The following will deeply explore the microscopic evolution mechanism of this non-equilibrium state.

**Fig 2:**
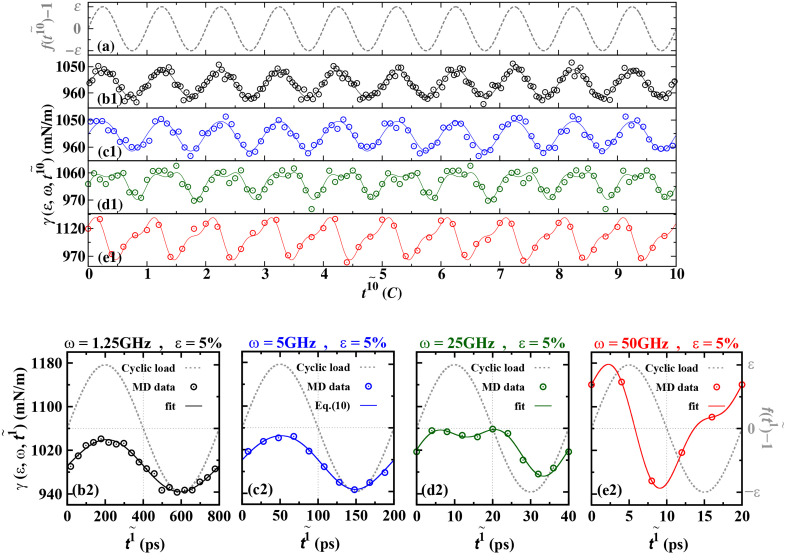
The LVI system of molten titanium (Ti) (*T *=* T*_m_* *= 1531 K) exhibits a steady-state oscillatory response of dynamic surface tension when subjected to sinusoidal cyclic loads with different loading frequencies and load frequencies [the gray dotted lines in Fig 2(a) and Fig 2(b)], as shown in the curves (b1)-(e1) in Fig 2(a) and (b2)-(e2) in Fig 2(b). The initial loading conditions are *ω* = 1.25 GHz, *ε* = 3% ((b1), (b2)); *ω* = 25 GHz, *ε* = 5% ((c1), (c2)); *ω* = 50 GHz, *ε* = 3% ((d1), (d2)); *ω* = 50 GHz, *ε* = 5% ((e1), (e2)). The statistical average of the dynamic oscillation of surface tension is represented by hollow circles. The solid lines representing the oscillation in (b1)-(e1) are fitted using equation (5), with the relaxation time on the horizontal axis $\tilde{{𝐭}^{\text{1}\text{0}}}(\tau =\tilde{t^{\text{10}})}$. The solid lines representing the oscillation in (b2)-(e2) are fitted using equation (6). The relaxation time on the horizontal axis is $\tilde{{𝐭}^{\text{1}}}(\tau =\tilde{t^{\text{1}}})$.


In Fig 2(a) and Fig 2(b), we present the time evolution characteristics of the steady-state oscillation of surface tension under different frequencies (1.25 GHz, 5 GHz, 25 GHz, 50 GHz) and 5% load amplitude conditions. Specifically, Fig 2 (b1) and Fig 2 (b2), Fig 2 (c1) and Fig 2 (c2), Fig 2 (d1) and Fig 2 (d2), Fig 2 (e1) and Fig 2 (e2) correspond to the response situations of the above four frequencies. In Fig 2(a), (b1)-(e1) of the oscillation’s solid line represent the fitting results using equation (5).

$$\frac{\gamma (\varepsilon ,\omega ,\tau )}{\gamma_{\text{0}}(\varepsilon ,\omega )}=\text{1}+\sum_{n=\text{1}}^{\text{2}}A_{n}(\varepsilon ,\omega )sin[\text{2}\pi n\omega \tau +\delta_{n}(\varepsilon ,\omega )]$$ (5)

Where, the relaxation time of the horizontal axis is $\tilde{t^{\text{1}\text{0}}}(\tau =\tilde{t^{\text{1}\text{0}}})$, and the statistical average yields the surface tension $\gamma (\varepsilon ,\omega ,\tilde{t^{\text{1}\text{0}}})$ of the dynamic oscillation, which is represented by hollow circles.

To enhance the time resolution capability, equation (5) is used to fit the single-cycle dynamic surface tension, with the relaxation time $\tilde{t^{\text{1}}}$($\tau =\tilde{t^{\text{1}}})$ on the horizontal axis. As shown in b2-e2 of Fig 2b, the vertical axis represents the dynamic surface tension $\gamma \left(\varepsilon ,\omega ,\tilde{t^{\text{1}}}\right)$. This single-cycle analysis helps to more accurately characterize the dynamic response characteristics under high-frequency loading.

As shown in Fig 2a-b, under cyclic loading, the dynamic surface tension *γ(ε,ω,τ)* of the LVI (load vibration-induced) system exhibits significant oscillatory behavior. Its amplitude, phase shift, and baseline value $\gamma_{\text{0}}$(*ε*,*ω*) all systematically change with the variation of the driving frequency. This phenomenon suggests that the dynamic response mechanism of the LVI system under cyclic loading may have similarities with the physical behavior of the classical driven oscillator model. Notably, by regulating the collective density distribution in the LVI action area, effective control of the dynamic surface tension can be achieved. This provides a new perspective for understanding the control mechanism of interface behavior under high-frequency loading. The comparative analysis in Fig 2a and 2b further reveals the intrinsic correlation between the multi-cycle statistical approach and the single-cycle high-resolution observational scale in terms of system response.

When the LVI system enters the steady state, the dynamic surface tension *γ*(*ε*,*ω*,*τ*) exhibits periodic oscillations around the reference value $\gamma_{\text{0}}$(*ε*,*ω*). The study found that the LVI system shows significant dynamic response characteristics to the cyclic load frequency *ω* and amplitude *ε*. At low frequencies of *ω* = 1.25 GHz and 5 GHz, the oscillation waveform of *γ*(*ε*,*ω*,*τ*) closely matches the sine waveform of the driving signal. However, as ω increases (*ω* = 25 GHz and 50 GHz), *γ*(*ε*,*ω*,*τ*) gradually exhibits nonlinear response characteristics. Its oscillation waveform deviates from the sine wave shape and additional frequency components appear. In Fig 2 (d1-d2) and (e1-e2), the surface tension deviates from the perfect sine oscillation and exhibits non-sinusoidal modal oscillation.

To deeply analyze this phenomenon, we conducted high-time-resolution (single-cycle scale) observations and found that the oscillation amplitude, phase shift, and reference value of *γ*(*ε*,*ω*,*τ*) all systematically changed with the driving conditions. This response characteristic is highly consistent with the prediction of the classical forced oscillator model [65], indicating that the collective density fluctuations in the LVI region are the intrinsic mechanism causing the dynamic changes in surface tension. It is worth noting that under a 50 GHz high-frequency drive, the dynamic surface tension of the titanium (Ti) melt exhibits a different response behavior from that reported for the lead (Pb) melt [46]. At low frequencies of *ω* = 1.25 GHz and 5 GHz, no biphase oscillation mode was observed in the melt. This difference reveals the material dependence of the low-frequency response characteristics of the metal liquid. At high frequencies of *ω* = 25 GHz and 50 GHz, the response characteristics of the melt show a multi-frequency oscillation form. Based on the forced oscillator theory, we used the Fourier series expansion method (considering the first two terms mainly) to quantitatively analyze the dynamic response of *γ*(*ε*,*ω*,*τ*). Through data fitting of equation (5), not only was the applicability of the theoretical model verified, but also it was revealed that under the conditions where the load frequency reached 50GHz and the load amplitude was as high as 5%, this model could accurately describe the intense oscillation phenomenon of the dynamic surface tension. Through the data fitting of equation (5), not only the applicability of the theoretical model was verified, but also it was revealed that under the conditions of a load frequency of 50 GHz and a load amplitude of up to 5%, this model could accurately describe the intense oscillation phenomenon of the dynamic surface tension.

**Fig 3:**
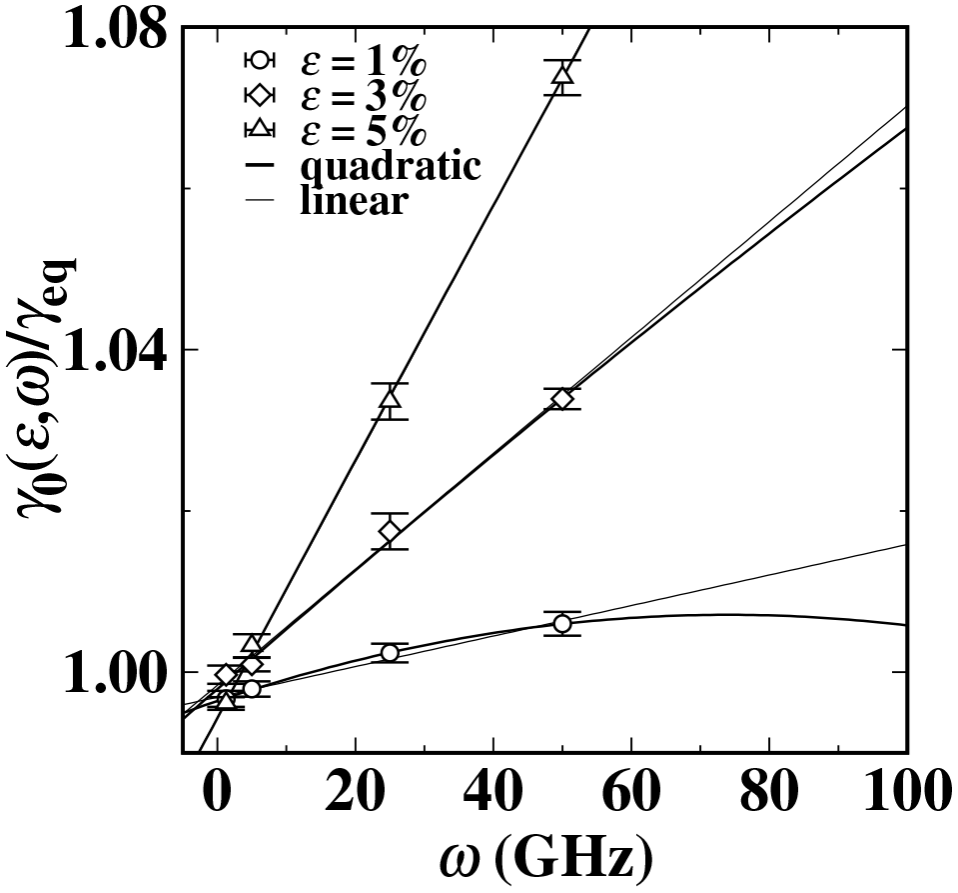
The average value of the dynamic surface tension $\gamma_{0}\left(\varepsilon ,\omega \right)$ of the titanium (Ti) liquid varies dynamically with the load frequency *ω* and the load amplitude *ε.* The light black and black solid lines are the results obtained by weighted least squares fitting using a linear function and a quadratic function, respectively.

As shown in Fig 3, we use the ratio $\gamma_{\text{0}}(\varepsilon ,\omega )/\;\gamma_{eq}$ to represent the variation of the dynamic surface tension $\gamma_{\text{0}}(\varepsilon ,\omega )$. When a fixed cycle load frequency *ω* or amplitude *ε* is applied, the surface tension of the titanium (Ti) liquid at the equilibrium state significantly increases. While keeping the applied cycle load amplitude *ε* unchanged, when the frequency is lower than 50 GHz, the dynamic surface tension $\gamma_{\text{0}}(\varepsilon ,\omega )$ shows a monotonically increasing trend. When the driving load frequency is 50 GHz and the load amplitude is 5%, the average dynamic surface tension of the Ti liquid is approximately 1069 mN/m. Compared to the surface tension at the equilibrium state, it increases by about 7%. Given that there is no quantitative theory that can describe the variation law of the dynamic surface tension mean $\gamma_{\text{0}}(\varepsilon ,\omega )$, we attempt to use the weighted least squares method to fit $\gamma_{\text{0}}(\varepsilon ,\omega )/\;\gamma_{eq}$ with linear and quadratic functions. From Fig 3, it is found that the dynamic surface tension of the Ti liquid more follows the quadratic function pattern of variation. For the result of the dynamic surface tension mean $\gamma_{\text{0}}(\varepsilon ,\omega )$ gradually increasing, this is not explainable by the classical forced damping vibration theory model [65] (the middle position of the oscillator remains unchanged). As we hypothesized, the growth of $\gamma_{\text{0}}(\varepsilon ,\omega )$ may be due to the change in the atomic packing structure near the LVI of the molten Ti under the cycle load, and the dynamic properties of the liquid-vapor interface also change accordingly. Regarding the underlying reason for this, it may be that the forced resonator is a single system, while the two liquid surface systems are composed of a large number of atoms, and the energy conversion characteristics of the stacking structure regulation in the multi-particle system are much more complex than those in the single-particle system. To deeply understand the fundamental reason for the response of the dynamic surface tension $\gamma_{\text{0}}(\varepsilon ,\omega )$ under the cycle load, we will analyze and interpret based on the calculated dynamic interface distribution data.

We will employ the forced vibration theory [65] to explain the periodic response behavior of the dynamic surface tension of the Ti liquid. When the oscillation system has a certain amplitude $A_{n}$ and phase $\delta_{n}$, the functional relationship between the load frequency *ω* and the load amplitude *ε* (the phase is independent of the load amplitude) is as shown in equations (6)-(7).


$$A_{n}^{\text{2}}(\varepsilon ,\omega )=\frac{f_{n}^{\text{2}}}{{\left(\text{4}\pi^{\text{2}}\omega_{\text{0}}^{\text{2}}-\text{4}\pi^{\text{2}}n^{\text{2}}\omega^{\text{2}}\right)}^{\text{2}}+\text{16}\pi^{\text{2}}\beta^{\text{2}}n^{\text{2}}\omega^{\text{2}}}$$ (6)

$$\delta_{n}(\omega )=\text{arctan}\left(\frac{n\beta \omega }{{\pi \omega }_{\text{0}}^{\text{2}}-\pi n^{\text{2}}\omega^{\text{2}}}\right)$$ (7)

Where, *ε* represents the applied amplitude, *ω* represents the applied frequency, *β* is the damping coefficient, *ω*_0_ is the inherent frequency of the liquid, and *n* = 1, 2 represent the two-order frequency vibration modes. It should be noted that based on the universality of the Fourier series theory, any periodic driving force system (such as *f*(*t*)) can be constructed through Fourier expansion, which is composed of a linear superposition of a series of sinusoidal driving force oscillations *f*_*n*_(*t*). Therefore, here *f*_*n*_ is the amplitudes of the two dominant Fourier components of the periodic driving force. In the actual data fitting, we found that due to the complex system of the titanium (Ti) liquid surface. When comparing with simple elastic dampers, directly applying equations (6–7) is difficult to describe the calculation results of *A*_*n*_ and *δ*_*n*_ well. Therefore, we conducted a global analysis of the dynamic surface tension data and reorganized and corrected the equations (6–7). The vibration modes of the *n* = 1 and *n* = 2 components respectively adopt different inherent frequencies *ω*_01_ and *ω*_02_ and different damping coefficients *β*_1_ and *β*_2_, and the specific forms are as shown in equations (8)-(9).


$$A_{n}^{\text{2}}(\varepsilon ,\omega )=\frac{f_{n}^{\text{2}}}{{\left(\text{4}\pi \omega_{\text{0}n}^{\text{2}}-{{\text{4}\pi }^{\text{2}}n}^{\text{2}}\omega^{\text{2}}\right)}^{\text{2}}+{\text{16}\pi }^{\text{2}}{\beta_{n}}^{\text{2}}n^{\text{2}}\omega^{\text{2}}}$$ (8)

$$\delta_{n}(\omega )=\text{arctan}\left(\frac{n\beta_{n}\omega }{{\pi \omega }_{\text{0}n}^{\text{2}}-{\pi n}^{\text{2}}\omega^{\text{2}}}\right)$$ (9)

**Fig 4:**
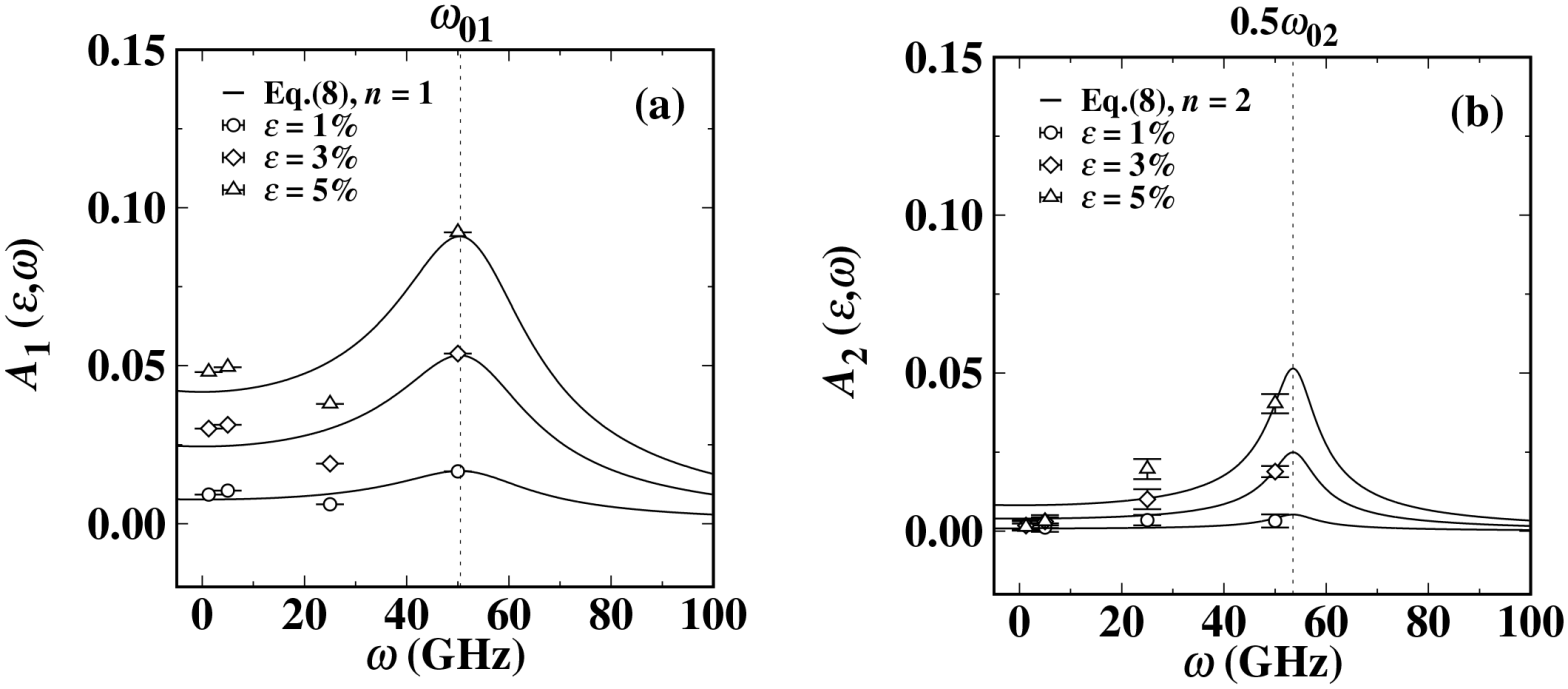
The response amplitudes ${𝐀}_{1}(\varepsilon ,\omega )$ and ${𝐀}_{2}(\varepsilon ,\omega )$ of the dynamic surface tension of titanium (Ti) liquid vary with the load frequency and load amplitude. The solid lines in (a) and (b) are the results of the global weighted least squares fitting of the amplitudes obtained from all data points to equation (8), with n being 1 and 2 respectively. The positions of the natural frequencies ($\omega_{\text{01}}$ and $\omega_{\text{02}}$) in the two figures are marked by vertical dashed lines.

Fig 4(a) and (b) present the response characteristics of the two key fluctuation amplitudes $A_{\text{1}}(\varepsilon ,\omega )$ and $A_{\text{2}}(\varepsilon ,\omega )$ of the dynamic surface tension, as fitted by Equation (8), with respect to the driving natural frequency ω under three different cyclic load amplitudes. As the load frequency and amplitude increase, both amplitudes $A_{\text{1}}(\varepsilon ,\omega )$ and $A_{\text{2}}(\varepsilon ,\omega )$ exhibit a significant growth trend. As shown in Fig 4, at higher driving load frequencies (50 GHz) and load amplitudes (*ε* = 5%), the amplitudes of the *n* = 1 component *A*_1_ and the *n* = 2 component *A*_2_ reach their maximum values. In Fig 4(a), the maximum amplitude *A*_1_ of the Ti liquid *n* = 1 component can reach 9.8% of *γ*_*eq*_. In Fig 4(b), the amplitude *A*_2_ of the *n* = 2 component can reach 4% of *γ*_*eq*_. When comparing these two amplitudes comprehensively, we find that the value of amplitude $A_{\text{2}}$(*ε*, *ω*) is generally lower than that of amplitude $A_{\text{1}}$(*ε*, *ω*). Especially in cases with lower driving load frequencies and amplitudes, the value of amplitude $A_{\text{2}}$(*ε*, *ω*) is almost negligible, approaching zero. This feature indicates that in low-frequency and low-amplitude conditions, the dynamic surface tension can maintain nearly perfect sinusoidal oscillation characteristics. The research results show that the oscillation behavior of the dynamic surface tension has significant similarity to the forced harmonic oscillator model in classical mechanics. This discovery provides an important basis for understanding its physical mechanism.

**Fig 5:**
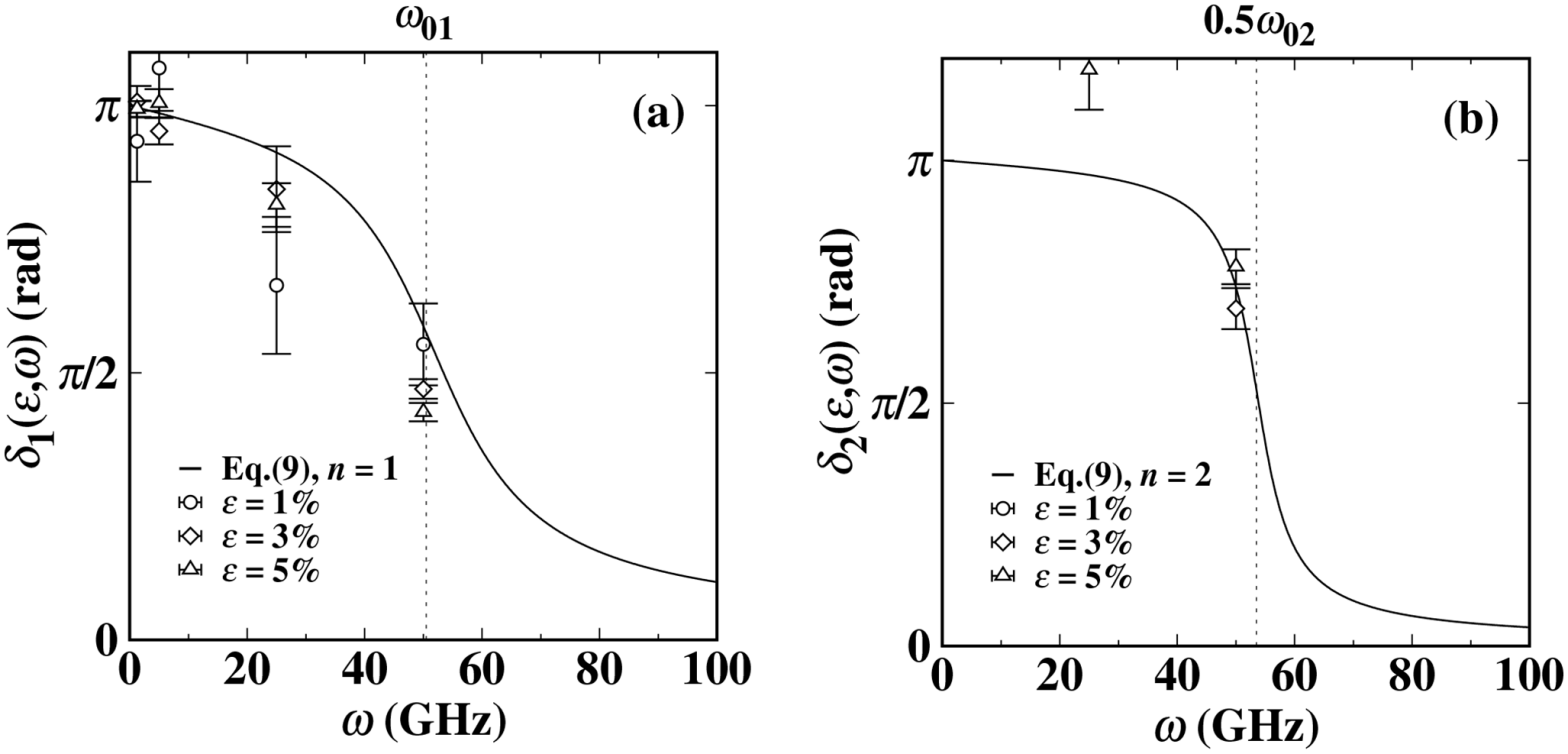
Under different loading amplitudes *ε*, the phase responses *δ*_1_ (*ω*) and *δ*_2_ (*ω*) of the dynamic surface tension of titanium (Ti) change with the cyclic loading frequency *ω.* The solid lines in (a) and (b) are the results of fitting equation (9) using the global weighted least squares method for the *n* = 1 and *n* = 2 modes, respectively. The resonance peak in (a) is significantly broader than that in (b), and only the data points with significant amplitude *A*_2_ (*ε*, *ω*) are selected. The positions of the system natural frequencies *ω*_01_ and *ω*_02_ are indicated by the vertical dashed lines.

Our experimental results indicate that the phase differences $\delta_{\text{1}}(\omega )$ and $\delta_{\text{2}}(\omega )$ generated by the dynamic surface tension both exhibit significant frequency dependence. Fig 5 is obtained by fitting with equation (9). It is worth noting that although Fig 4(b) has confirmed the existence of a finite magnitude for *A*_2_ (*ε*, *ω*), due to its relatively smaller increase compared to *A*_1_ (*ε*, *ω*) (especially in the low-frequency region, the value of *A*_2_ approaches zero), the phase difference *δ*_2_ (ω) has a large degree of uncertainty. Further research reveals that, as predicted by equation (9), the values of phase differences *δ*_1_ (ω) and *δ*_2_ (ω) under different driving frequencies are basically unaffected by the amplitude of cyclic loading. As the driving frequency continues to increase, the measurement error of phase difference *δ*_2_ (*ε*, *ω*) significantly increases. This leads to a significant decrease in the data reliability. It is particularly noteworthy that in the low-frequency region (*ω* is small), *δ*_1_ (*ω*) approaches zero. This phenomenon clearly indicates that the oscillation of the dynamic surface tension at this time is nearly perfectly synchronized with the cyclic load. This characteristic is intuitively verified in the experimental results shown in Fig 2a (b1) and Fig 2b (b2). When applying different load frequencies *ω* to drive the liquid system, if the dynamic surface tension response at the interface lags behind the externally applied load oscillation, the phase difference *δ*_1_ (*ω*) will be less than 90 degrees, or even almost zero. This phenomenon suggests that the dynamic surface tension oscillation of the Ti liquid at low frequencies may follow an oscillation mode highly consistent with the load driving form, as shown in Fig 2a (b1) and Fig 2b (b2). However, at high frequencies, the dynamic surface tension exhibits multi-frequency oscillation modes, as shown in Fig 2a (e1) and Fig 2b (e1).

The comparative analysis of the experimental data with the theoretical model indicates that equations (8) and (9) can accurately describe the key characteristic parameters of the dynamic surface tension of the Ti liquid. As shown by the solid lines in Figs 4 and 5, there is a high degree of consistency between the theoretical predictions and the measured amplitude $A_{n}(\varepsilon ,\omega )$ and phase difference $\delta_{\text{n}}(\varepsilon ,\omega )$, verifying the reliability of this model in characterizing the dynamic surface tension behavior of liquid metals. For example, through data fitting, we obtained the following estimated values: $\omega_{\text{01}}$=53.4(5) GHz, $\omega_{\text{02}}$=107.7(4) GH, $\beta_{\text{1}}$=12.6(1) GHz, and $\beta_{\text{2}}$=8.6(2) GHz. These results reveal that by estimating $\beta_{\text{1}}/\omega_{\text{01}}\approx$0.235 and $\beta_{\text{2}}/\omega_{\text{02}}\approx \text{0}.\text{080}$, we can infer that both of these components systems are in an underdamped state. When the driving frequency is close to the natural frequencies *ω*_01_ and *ω*_02_ of the system, the two amplitudes *A*_1_ (*ε*, *ω*) and *A*_2_ (*ε*, *ω*) of the dynamic surface tension oscillations will significantly increase and may reach their respective resonance peaks. Specifically, when the cyclic loading frequency *ω* is set to 50 GHz, which is very close to the driving frequency *ω*_01_, and this frequency is significantly different from the natural frequency *ω*_02_ of the *n* = 2 component. Therefore, the *n* = 2 component term in Fig 4(b) does not reach the resonance state. However, *β*_1_/*ω*_01_ is three times that of *β*_2_/*ω*_02_, which leads to the *n* = 2 component of the dynamic surface tension oscillation being weaker compared to the *n* = 1 component. Its amplitude peak becomes more concentrated, and the peak width narrows, and the second component (*n* = 2) of the dynamic surface tension oscillation has a steeper phase attenuation slope than the first component (*n* = 1). This is consistent with the data shown in Figs 4 and 5. However, $\beta_{\text{1}}/\omega_{\text{01}}$ is three times that of ${\;\beta }_{\text{2}}/\omega_{\text{02}}$, this significant difference leads to the *n* = 2 oscillation component of the dynamic surface tension being relatively weaker than the *n* = 1 component. Specifically, there are three characteristic changes: (1) The amplitude peak distribution of the *n* = 2 component is more concentrated; (2) The resonance peak width of the *n* = 2 component is significantly narrower; (3) The slope of the phase attenuation of the *n* = 2 component is steeper than that of the *n* = 1 component. Due to the weak amplitude of the *n* = 2 component in the titanium system, the intensity of the drastic phase attenuation of the second harmonic cannot fully match the predicted results, which is consistent within the error range. These characteristics are fully consistent with the measured data in Figs 4 and 5. This confirms that the dynamic surface tension oscillation of the *n* = 1 component and the *n* = 2 oscillation component of the titanium system have significant differences in their dynamic behaviors. For the study of the titanium system, more data may be needed to understand and determine the range of multi-frequency modes.

Currently, the liquid surface is typically modeled as an elastic membrane system for study [66]. Based on this theoretical framework, this research further explores the physical meanings and intrinsic correlations of the above-mentioned fitted parameters including *ω*_01_, *ω*_02_, *β*_1_, and *β*_2_. The dynamic surface tension characteristics of liquid titanium are closely related to the non-equilibrium evolution process of the surface atomic microstructure. Through experimental measurement and theoretical analysis, we obtained two characteristic natural frequencies *ω*_01_ (53.4 ± 5 GHz) and *ω*_02_ (107.7 ± 4 GHz). They correspond to characteristic time scales of 4.68 ps and 2.32 ps respectively. It is worth noting that each complete oscillation cycle contains two ascending stages and two descending stages, and this periodic behavior may have a direct correlation with the relaxation process of atomic density fluctuations. Based on the calculation analysis of the dynamic structure factor, we found that the dynamic behavior of the near-surface atomic layer of liquid titanium has significant characteristics. By using the method of the main peak’s inverse half-width of the dynamic structure factor [67], we determined the characteristic relaxation time of particle dynamics. The research results show that the longitudinal collective dynamics of the near-surface atomic layer significantly slows down, and its density relaxation time is at least doubled. This phenomenon is consistent with the reports of Reichert et al. [68] and Del Rio et al. [69]. Particularly noteworthy is that the time scales corresponding to *ω*_01_ and *ω*_02_ (4.68 ps and 2.32 ps) are approximately 8 times and 4 times the characteristic relaxation time of the bulk liquid phase (0.56(4) ps). This large difference reveals that both the bulk liquid body and the surface region are affected, with the surface having a greater influence. The abnormal long time scale of *ω*_01_ suggests that the surface tension oscillations may have spatial inhomogeneity, and the response of the near-surface layer and the sub-surface layer to dynamic loads is different. These findings indicate that under ultra-short-time loading, the near-surface atomic structure of liquid titanium will be significantly disturbed, and it exhibits a slow recovery dynamics after the removal of the load. This phenomenon indicates that the traditional bulk liquid phase theory may not be able to fully explain the dynamic behavior of the liquid metal surface, and a new theoretical framework needs to be established. Moreover, regarding the influence mechanism of damping constants *β*_1_ and *β*_2_ on the dynamic surface tension, further experimental research and theoretical discussion are still needed.

**Fig 6:**
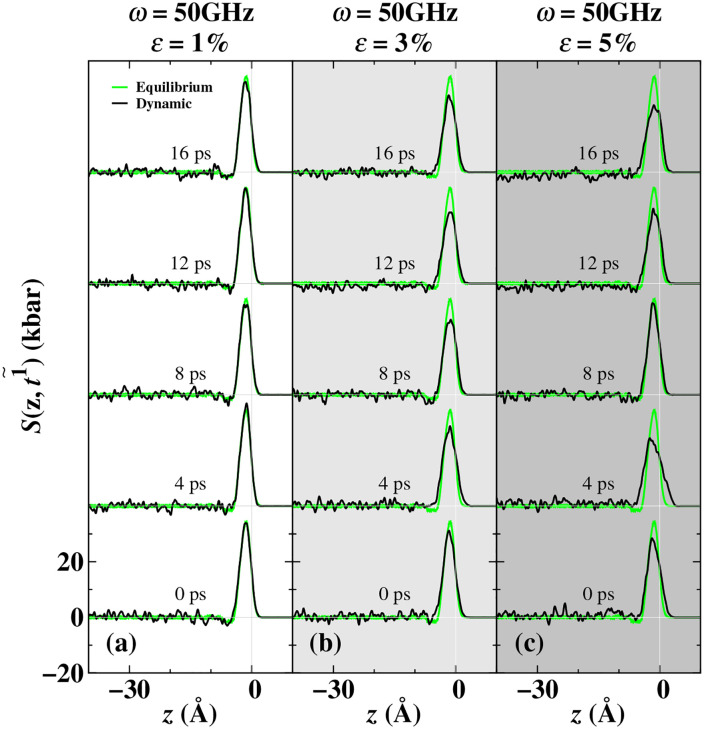
It shows the periodic evolution of the fine-grained stress distribution of molten Ti. At 50 GHz, stress distribution results at different times were obtained by adjusting the load at 1% (figure a), 3% (figure b), and 5% (figure c). *ω* represents the load frequency, and *ε* represents the load amplitude. The green curve represents the fine-grained stress distribution at the melting point in the equilibrium state. The black curve represents the fine-grained stress distribution after periodic averaging. The horizontal gray thin line represents the plateau value of the internal stress in the block, and the vertical gray thin line represents the interface position.

To deeply explore the microscopic mechanism of dynamic surface tension during the driven oscillation process, this study systematically investigated the temporal and spatial evolution laws of the dynamic fine-grained stress field and density field on the molten Ti surface under cyclic loading. As shown in Fig 6, the microscopic stress oscillation behavior was analyzed through the difference between the dynamic tangential pressure component and the normal pressure component. As shown in Fig 6, at a driving frequency of 50 GHz, the distribution of the dynamic fine-grained stress *S*(*z*,$\tilde{t^{\text{1}}})$ on the molten Ti surface exhibited dynamic evolution characteristics with the increase of the load amplitude *ε*. When *ε* = 1%, only a slight adjustment of the positive stress peak on the surface layer occurred. As *ε* increased to 3%−5%, the stress distribution gradually became unstable, showing a broadening of the peak and a reduction in the amplitude fluctuation. This change may have the effect of weakening the damping effect of the liquid layer below the surface. In the region behind the positive stress peak of the liquid, the mean stress showed overall fluctuations up and down. In the Ti liquid system, the negative stress peak increased less with the increase of the driving amplitude. At higher loads of *ε* ≥ 3%, sub-surface stress was observed to significantly contribute to the dynamic surface tension, and its mechanism was different from the adjustment of the surface layer structure and corresponded to the response function of *n* = 2 component. This reveals the complex characteristics of multi-scale nonlinear coupling of surface and sub-surface stress under high load conditions.

For the Ti liquid, the internal stress within the blocks at the 12th ps with a load amplitude of 3% and at the 12th and 16th ps with a load amplitude of 5% was all lower than 0 kbar. However, the internal stress within the blocks at the 0th and 4th ps with a load amplitude of 5% was all higher than 0 kbar. These indicate that in the static hydrostatic equilibrium state of the fluid, the stress in the block region of the molten Ti liquid is zero. During dynamic loading, the stress field shows significant spatial heterogeneity: the zero-stress area corresponds to the non-static pressure state of the fluid, and the positive/negative stress areas respectively represent local tensile/compressive states. Fig 6 shows that the dynamic stress curve exhibits a decay-oscillating relaxation characteristic after reaching the peak, and the block region of the molten Ti liquid continuously shows a non-zero stress distribution. The alternating distribution of positive and negative stress intuitively reveals the dynamic stretching-compression process, which contrasts strongly with the balanced state of zero stress. This verifies the non-equilibrium effect induced by cyclic loading.

According to the classical density functional theory [70], the free energy field of the system is directly related to the liquid density distribution. The steady-state atomic configuration and density distribution of the liquid system are determined by the minimum state of the system’s global free energy. Based on this theoretical framework, we believe that when a cyclic load is applied to the molten Ti liquid, it will cause the density distribution of the local liquid region to deviate from its equilibrium state. However, in this non-equilibrium state, the system will achieve a reduction in free energy by spontaneously adjusting its density field distribution, thus approaching a new stable state. At lower load frequencies (such as *ω* = 1.25 GHz), even with a 5% load amplitude, since the local liquid density regulation process induced by the cyclic load is relatively slow, its response time scale (*C*/4 = 200 ps) is more than three orders of magnitude longer than the intrinsic density relaxation time of the liquid Ti [0.56(4) ps]. Therefore, in this case, the dynamic fine-grained density distribution and stress state of the surface region remain basically consistent with the equilibrium state.

In contrast, under the higher load frequency condition of *ω* (50 GHz), the time scale of the cyclic load (*C*/4 = 5 ps) is closer to the relaxation time of the liquid density. This results in an anisotropic relaxation process of atomic rearrangement. This dynamic competition mechanism causes significant adjustments to the surface fine-grained density and stress distribution, and generates non-fluid static pressure within the liquid. Due to the extremely short duration of the high-frequency cyclic load, the fine-grained density field may not fully relax to the equilibrium state but is instead frozen in a metastable state with relatively lower free energy. This mechanism may partially explain the experimental phenomenon that the average dynamic surface tension of molten Ti liquid increases after reaching the steady state.

**Fig 7:**
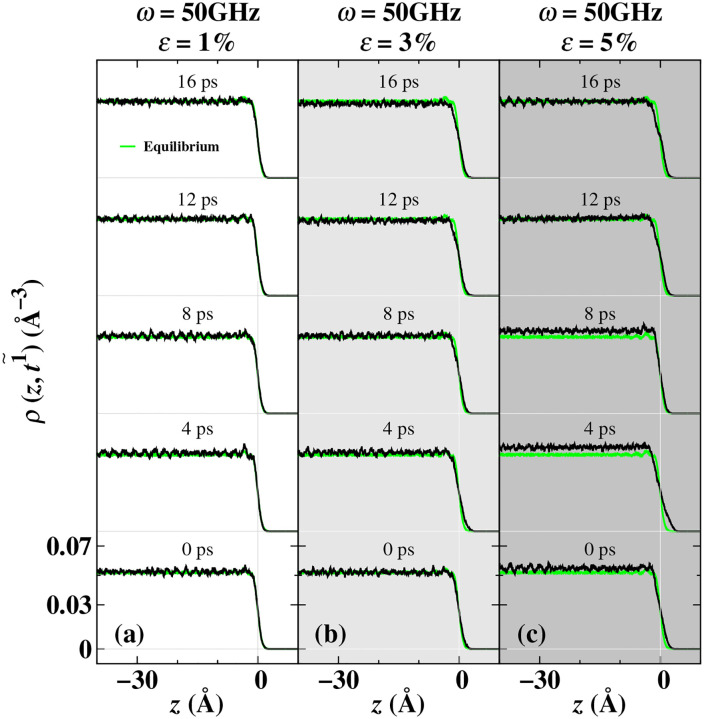
It demonstrates the periodic evolution of the fine-grained density distribution of the Ti liquid. At 50 GHz, different density distribution results at different times were obtained by adjusting the load at 1% (figure a), 3% (figure b), and 5% (figure c). *ω* represents the load frequency, and *ε* represents the load amplitude. The green curve represents the fine-grained density distribution at the melting point in the equilibrium state. The black curve represents the fine-grained density distribution after periodic averaging. The horizontal gray thin line represents the zero-density of vacuum, and the vertical gray thin line represents the interface position.

Based on equation (2), we calculated the fine-grained dynamic *ρ*(*z*,$\tilde{t^{\text{1}}}$) distribution shown in Fig 7, which enables us to discover more details about the local atomic dynamic adjustments. For instance, at the load frequency *ω* (50 GHz) and load amplitude *ε*(3%, 5%), the density in the sub-surface block region shows a uniform increase or decrease. These changes have deviated from the equilibrium value of the liquid phase density, and the outermost region becomes wider. This indicates that the dynamic adjustments in the sub-surface region are different from those in the outermost region, which is consistent with the previous changes in the dynamic stress distribution adjustment at the load amplitudes of *ε* = 3% and 5%.

**Fig 8:**
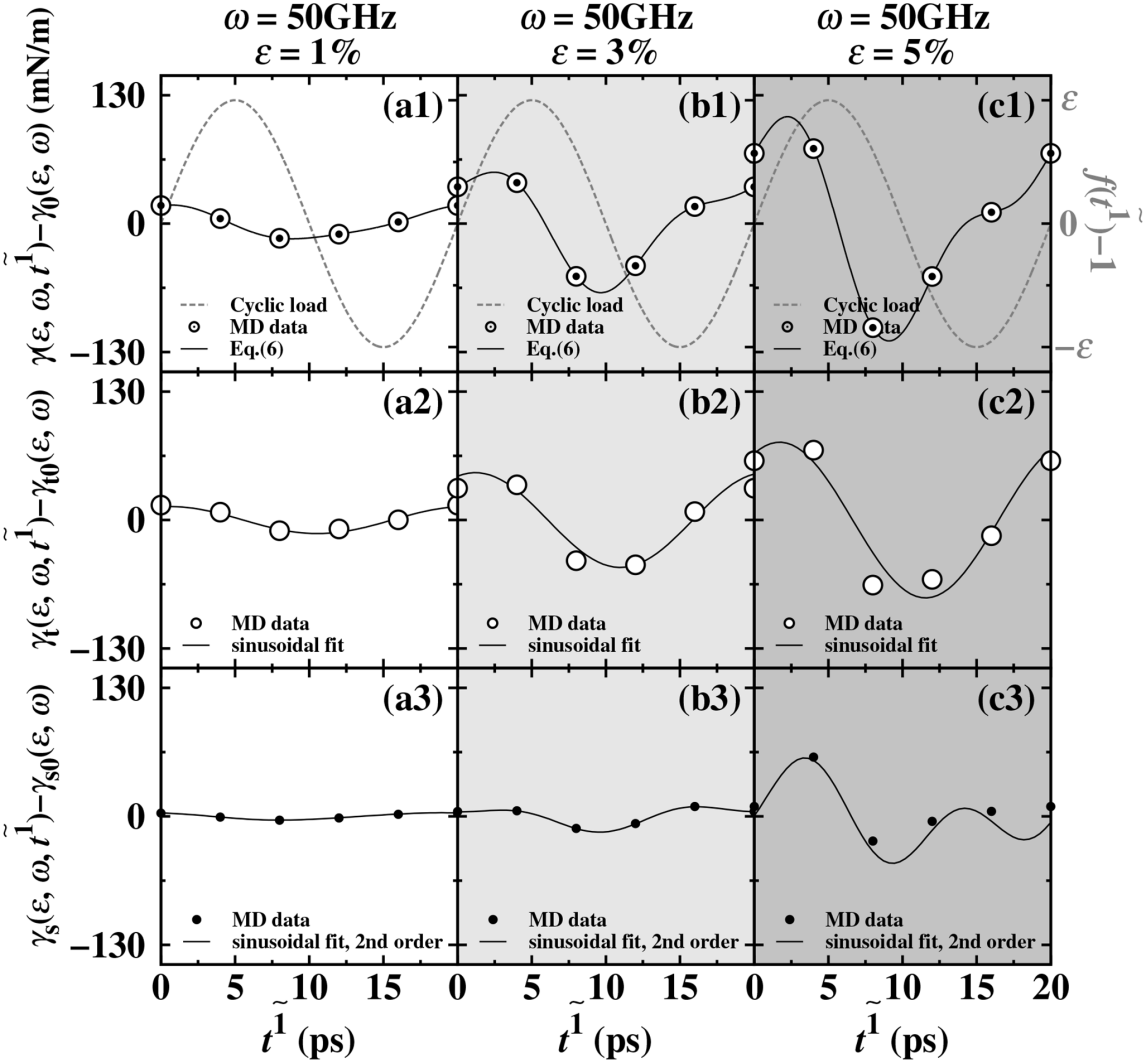
For the contribution results of the stress at a cycle frequency of 50 GHz and load amplitudes of 1% (a1-a3), 3% (b1-b3), and 5% (c1-c3), the white and solid circles represent the outermost positive peak of the dynamic surface stress distribution and the stress contribution in the remaining areas, respectively.

To obtain further quantitative evidence, we separately calculated the local stress contributions of the outermost layer and the sub-surface layer [40,41]. For the surface region of the Ti liquid, it can be defined as the area corresponding to the first positive peak of the stress. According to Fig 6, for the stress distribution of the Ti liquid, the outermost layer region spans a size of approximately 3 nm, and the remaining stress regions are all sub-surface stress regions. Thus, the dynamic surface tension is decomposed into the contributions of the outermost layer and the sub-surface layer through equation (4) for analysis. $\gamma (\tau )=\gamma_{t}(\tau )+\gamma_{s}(\tau )=\int_{z_{\text{lo}}}^{z_{\text{1}}(\tau )}\;S(z,\tau )dz+[\int_{z_{\text{1}}(\tau )}^{z_{hi}}\;S(z,\tau )dz-S_{l}(\tau )L_{l}(\tau )]$. $\gamma_{t}(\tau )$ and $\gamma_{s}(\tau )$ are the stress contributions of the outermost layer and other regions of the stress distribution, respectively. We analyzed the stress contribution decomposition results of the dynamic surface tension $\gamma (\varepsilon ,\omega ,\tilde{t^{\text{1}}})(\tau =\tilde{t^{\text{1}}})$ obtained under the cyclic load frequency of 50 GHz, getting the outermost layer stress contribution $\gamma_{t}(\varepsilon ,\omega ,\tilde{t^{\text{1}}})$ and the sub-surface contribution $\gamma_{s}(\varepsilon ,\omega ,\tilde{t^{\text{1}}})$. We used equation (5) to fit the dynamic surface tension $\gamma (\varepsilon ,\omega ,\tilde{t^{\text{1}}})(\tau =\tilde{t^{\text{1}}})$, the outermost contribution $\gamma_{\text{t}}(\varepsilon ,\omega ,\tilde{t^{\text{1}}})$ and the sub-surface contribution $\gamma_{\text{s}}(\varepsilon ,\omega ,\tilde{t^{\text{1}}})$. $\gamma (\varepsilon ,\omega ,\tilde{t^{\text{1}}})$ obtained a basic constant $\gamma_{\text{0}}(\varepsilon ,\omega )$. For $\gamma_{t}(\varepsilon ,\omega ,\tilde{t^{\text{1}}}\backslash textrm\mathrm{and}\;\gamma_{\text{s}}(\varepsilon ,\omega ,\tilde{t^{\text{1}}})$, equation (5) can also be used for fitting. $\gamma_{t}(\varepsilon ,\omega ,\tilde{t^{\text{1}}}\backslash textrm\mathrm{and}{\;\gamma }_{\text{s}}(\varepsilon ,\omega ,\tilde{t^{\text{1}}})$ respectively obtained a basic constant $\gamma_{t\text{0}}(\varepsilon ,\omega$and $\gamma_{s\text{0}}(\varepsilon ,\omega )$. After calculation, the equation $\gamma_{\text{0}}(\varepsilon ,\omega )=\gamma_{t\text{0}}(\varepsilon ,\omega )+\gamma_{s\text{0}}(\varepsilon ,\omega )$ was obtained. Based on this, we calculated $\gamma (\varepsilon ,\omega ,\tilde{t^{\text{1}}})-\gamma_{\text{0}}(\varepsilon ,\omega )$, getting the points shown in the circles in Fig 8 (a1-c1). We used equation (5) again for fitting, and the solid lines shown in Fig 8 (a1-c1) were obtained. $\gamma_{t}(\varepsilon ,\omega ,\tilde{t^{\text{1}}})-\gamma_{t\text{0}}(\varepsilon ,\omega )$ and $\gamma_{s}(\varepsilon ,\omega ,\tilde{t^{\text{1}}})-\gamma_{s\text{0}}(\varepsilon ,\omega )$ were respectively the white circles in Fig 8 (a2-c2) and the black points in Fig 8 (a3-c3). Both were fitted again using equation (5), and the black solid lines in Fig 8 (a2-c2) and Fig 8 (a3-c3) were obtained.

Based on the reduced oscillation results shown in Fig 8 (a1-c1), under the condition of smaller amplitude (*ε* = 1%), the dynamic surface tension exhibits a typical single-frequency sinusoidal oscillation. This is only present in the fundamental frequency n = 1 mode, corresponding to the fitting equation (5) with *n* = 1. However, in Fig 8 (a2-c2), the contribution of the outermost stress region does not show non-sinusoidal oscillation. Further decomposition of the stress distribution study indicates that the contribution of the positive peak of the outermost stress (Fig 8 a2-c2) maintains good sinusoidal characteristics under different amplitudes, and its oscillation behavior is mainly dominated by the sinusoidal mode (*n* = 1). Even at high amplitudes (*ε* = 3% and 5%), no obvious harmonic components are observed. The reduced results of the sub-surface stress contribution (Fig 8 a3-c3) show that Fig 8 (b3) presents a nearly sinusoidal oscillation characteristic. This indicates that the dynamic response of the sub-surface may also be determined solely by the sinusoidal mode (*n* = 1).

However, Fig 8 (b3) shows a non-sinusoidal oscillation characteristic. It is noteworthy that, through the observations in Fig 8, we find that the outermost liquid stress region of the Ti liquid contributes mainly to the dynamic surface tension, while the sub-surface stress contribution plays a fundamental role, and the sub-surface contribution affects the fluctuation pattern of the dynamic surface tension. The oscillation frequencies *ω*_01_ (*n* = 1) and *ω*_02_ (*n* = 2) of the dynamic surface tension may be related to the characteristic frequencies of the outermost and sub-surface stress responses. These microscopic anatomical data support the above-mentioned dynamic interface density and stress distribution of the molten Ti liquid and the inferred observation results. It is concluded that the sub-surface may have the same two natural frequencies as driven by cyclic loads. However, the outermost layer may respond to the applied load with the same dynamic surface tension oscillation behavior at the same frequency. Based on the existing data, we can only speculate that there may be a coupling relationship between the two, and the specific mechanism still needs to be further verified by subsequent studies combined with more systematic experimental or simulation data.

## 5 Conclusion

This study employed the MD simulation method to systematically investigate the dynamic response behavior of the liquid-gas interface of molten metal Ti under unidirectional (*x*-direction) cyclic loading. By analyzing the stress distribution at the interface, the spatiotemporal evolution laws of the surface structure under different load frequencies and amplitudes were revealed, as well as its regulatory effect on the dynamic surface tension (excess stress). Based on this, we developed a comprehensive research method integrating MD simulation, dynamic characterization of interface microstructure, and Gibbs excess quantity calculation. This method can simultaneously capture the evolution of interface structure and the macroscopic surface tension response, and is suitable for studying the dynamic surface properties of metal liquid systems under non-equilibrium conditions. This not only overcomes the limitations of the traditional IK method in non-equilibrium conditions but also provides an effective means for understanding the regulatory mechanism of interface thermodynamic behavior under external field loading.

Research findings show that under transverse cyclic loading conditions, the liquid surfaces of Ti and Pb both exhibit obvious dynamic surface tension oscillations, and exhibit typical under-damping characteristics. The mean value of dynamic surface tension significantly increases with the increase of driving frequency and load amplitude, demonstrating a universal law across different systems. Meanwhile, the study also observed material-dependent response differences. There are significant differences in the transient peak and valley values and mean amplitudes of different systems, reflecting the intrinsic relationship between the microscopic structure evolution of the interface and the macroscopic response. This law provides an important theoretical basis for understanding the behavior of liquid interfaces in non-equilibrium states.

Based on the self-developed simulation, characterization and computation research system, we first achieved the dynamic sampling characterization of the surface tension of liquid Pb [46]. Then, we applied it to the liquid Ti system. The system investigated the steady-state oscillation and periodic response patterns of the dynamic surface tension under different load conditions. Due to the limitations of the traditional IK method in calculating non-equilibrium surface tension, we redefined the expression of instantaneous dynamic surface tension and accurately calculated it using the Gibbs excess method. Combining the forced vibration theory with Fourier series analysis, we fitted the instantaneous surface tension data under different conditions and obtained key parameters such as the mean surface tension, amplitude, phase difference, natural frequency, and damping coefficient.

During the steady-state oscillation phase, the response pattern of the dynamic surface tension to the periodic cyclic load is in accordance with the classical forced-damping vibration theory. However, the Ti liquid surface system exhibits a unique phenomenon. The mean value of its dynamic surface tension is significantly increased during the steady-state oscillation, and deviates from the equilibrium value without load. At the highest frequency (50 GHz) and amplitude (5%), the mean value of the dynamic surface tension is approximately 7% higher than the equilibrium state. And the peak and valley values are 9.8% and 4% higher and lower than the equilibrium surface tension, respectively. The research further reveals that this control behavior is material-dependent. Although the surface tension of different metal systems can be regulated through cyclic loading, the increase in the surface tension of the Ti liquid (about 7%) is higher than that reported in reference [46] for the Pb system (about 5%). This reflects the significant differences in the response of different metals to external dynamic excitation. These results not only reveal the dynamic behavior characteristics of the Ti liquid under cyclic loading, but also clarify its similarities and differences with the classical vibration theory.

From the dynamic surface tension results we calculated, we effectively extracted two generalized natural frequencies and two generalized damping constants. The experimental results indicate that the interface system is in an underdamped state, and it is predicted that resonance phenomena may occur at the highest driving frequency of 50 GHz. Through in-depth analysis of the fine-grained dynamic interface stress and dynamic density distribution, we found that the particle packing adjustment of the outermost surface and sub-surface of the Ti interface system is not completely synchronized with the load-driven response. This leads to differences in the contribution of the outermost layer and sub-surface to the dynamic surface tension response.What is even more interesting is that when analyzing non-equilibrium state elastic damping, it was found that the predicted values of this model ($\beta_{\text{1}}/\omega_{\text{01}}\approx$0.235 and ${\;\beta }_{\text{2}}/\omega_{\text{02}}\approx$0.080) were consistent with the experimental values from reference [44] ($\beta_{\text{1}}/\omega_{\text{01}}\approx$0.100 and $\;\beta_{\text{2}}/\omega_{\text{02}}\approx$0.005). Both sets of values indicated that cyclic loading would induce elastic damping oscillations in both systems. Although there was little difference in the damping values of the *n* = 1 mode between the two studies, there was a difference of nearly ten orders of magnitude in the *n* = 2 mode. This significant difference is likely due to the synergistic effect of the dynamic reconfiguration of interface atoms and the non-equilibrium effect. The non-fluid static pressure effect caused by the load impact led to periodic fluctuations in the internal density and stress of the liquid, and changed the particle packing structure at the interface. Through the analysis of the liquid surface layer (*n* = 1) and the sub-surface layer (*n* = 2), we discovered the contribution of the stress of different layers of the liquid to the dynamic surface tension. The stress of the surface layer played a basic contribution role, while the stress of the liquid layer below the surface played the main contribution role. Moreover, it could also change the form of the fluctuation of surface tension. Although the response of the stress contribution of the outermost layer and sub-surface and the response of dynamic surface tension have the same generalized natural frequency, no higher-frequency oscillatory response frequency was observed. Therefore, we speculate that although the outermost surface layer and sub-surface have the same natural frequency, this may be related to the microscopic time-scale changes in density relaxation caused by the load-driven in the corresponding regions. Although the loading frequency of 50 GHz is higher than that of conventional mechanical loading, it is comparable to ultrafast processes such as the propagation of shock waves induced by femtosecond lasers [47] and the lattice vibration relaxation during high strain rate deformation [71]. The correspondence of these time scales indicates that the interface dynamic behavior revealed in this study has significant reference value for understanding the non-equilibrium response of materials under extreme conditions. However, the current data is not sufficient to support reasonable speculation about the nature of the damping constants, and further exploration and more data are needed to deeply understand these phenomena.

Under cyclic loading conditions, the dynamic rearrangement of atoms in the interface region and the non-equilibrium accumulation behavior are the direct causes of the variation in macroscopic-scale dynamic surface tension. By analyzing the evolution laws of density distribution and stress field under different loading conditions, it was found that the oscillatory characteristics of dynamic surface tension and the change in the ordered degree of particle arrangement in the interface layer show a synchronous response. This macroscopic response and the quantitative correlation with the evolution of microstructure reveal the regulatory essence of the thermodynamic behavior of liquid interfaces under external fields. This provides a mechanistic explanation for understanding non-equilibrium interface phenomena. Meanwhile, different materials exhibit unique response characteristics. The specific manifestation is that parameters such as the transient peak and valley values of dynamic surface tension, as well as the mean increase, show regular differences among different systems. This discovery indicates that although non-equilibrium interface behavior follows common physical laws, its specific manifestation is still regulated by the inherent characteristics of the materials. This provides a scientific basis for targeted design of interface properties.

Surface tension, as a key parameter influencing droplet nucleation [72], wetting [73], spreading [74], and metal pre-melting [75,76] in fluid dynamics and capillary phenomena, its dynamic variation pattern plays a decisive role in understanding the mechanism of microscopic particle aggregation. This study reveals the regulatory mechanism of dynamic surface tension under load-driven conditions, providing a new theoretical perspective for optimizing material surface properties. To achieve precise regulation of liquid surface tension and optimize related material systems, it is necessary to establish a quantitative theoretical model that includes the inherent frequency, damping characteristics, and particle arrangement structure under steady-state oscillation of the liquid.

The theoretical outcomes of this study demonstrate broad prospects for industrial applications. In the field of metal casting, it can guide the precise regulation of melt flow behavior, thereby improving the surface quality and dimensional accuracy of castings. In additive manufacturing (3D printing) technology, it provides theoretical support for the optimization of the metal powder melting and solidification process, enhancing the surface morphology and mechanical properties of the formed parts. In laser processing, it helps predict and optimize the dynamic behavior of the melt pool, improving processing accuracy and surface finish. Moreover, this research can also extend to cutting-edge fields such as microfluidic device development and space material preparation, laying a scientific foundation for the intelligent regulation of fluid interface behavior. These potential applications not only demonstrate the engineering value of this study but also open up new avenues for a deeper understanding of material surface behavior. Through systematic research on surface tension characteristics and their regulation mechanisms, we hope to achieve precise control of fluid dynamic behavior and promote technological innovation in related fields.
